# The Anti-Inflammatory, Immunomodulatory, and Pro-Autophagy Activities of Probiotics for Colorectal Cancer Prevention and Treatment: A Narrative Review

**DOI:** 10.3390/biomedicines13071554

**Published:** 2025-06-25

**Authors:** Beatrice Garavaglia, Letizia Vallino, Alessandra Ferraresi, Annalisa Visciglia, Angela Amoruso, Marco Pane, Camelia Munteanu, Ciro Isidoro

**Affiliations:** 1 Laboratory of Molecular Pathology, Department of Health Sciences, Università del Piemonte Orientale, Via P. Solaroli 17, 28100 Novara, Italy; letizia.vallino@uniupo.it (L.V.); alessandra.ferraresi@med.uniupo.it (A.F.); 2 Probiotical Research Srl, Via E. Mattei 3, 28100 Novara, Italy; a.visciglia@probiotical.com (A.V.); a.amoruso@probiotical.com (A.A.); m.pane@probiotical.com (M.P.); 3 Department of Plant Culture, Faculty of Agriculture, University of Agricultural Sciences and Veterinary Medicine, 400372 Cluj-Napoca, Romania; camelia.munteanu@usamvcluj.ro

**Keywords:** butyrate, APC, autophagy, inflammation, microbiota, cytokines, cancer

## Abstract

Colorectal cancer (CRC) is a major global health concern, particularly in Western countries where there is high consumption of processed food. Gut microbiota, intestinal inflammation, and autophagy play pivotal roles in CRC initiation and progression. Probiotics and probiotic metabolites (particularly short-chain fatty acids) have emerged as potential preventive and adjuvant therapeutics by restoring a balanced gut microbiota, dampening inflammation, stimulating immune response, and improving barrier integrity and intestinal epithelial homeostasis by modulating autophagy. This narrative review discusses the current evidence supporting the anti-inflammatory, immunomodulatory, and pro-autophagy effects of probiotics and their metabolites and explores their potential preventive and therapeutic applications in CRC management.

## 1. Introduction

### 1.1. Colorectal Cancer: Epidemiology, Risk Factors, Prevention, and Therapy at a Glance

Worldwide statistics indicate that colorectal cancer (CRC) incidence ranks third and mortality second (after lung) in 2022, being now the first-leading and second-leading cause of cancer death in men and women, respectively [1]. The most significant risk factors for developing CRC are genetic predisposition (inherited mutations in relevant genes), a family history of CRC, and chronic inflammatory conditions of the gastrointestinal tract [2,3]. Additional factors associated with an increased risk of developing sporadic CRC include age over 50, male sex, obesity, and a sedentary lifestyle [4].

Surgery remains the gold standard treatment for CRC, although, in most cases, it is preceded or followed by adjuvant chemotherapy and radiotherapy to shrink or stabilize the tumor [5,6]. Standard chemotherapy protocols include monotherapy with 5-fluorouracil (5-FU) and multi-drug therapy combining 5-FU with leucovorin and oxaliplatin or with leucovorin and irinotecan [5]. More recently, novel strategies such as target therapy, gene therapy, and immunotherapy have been introduced to treat CRC [7]. Early detection of CRC through colonoscopy and modification of lifestyle risk factors are crucial for prevention and reducing the risk of developing the tumor [8]. The highest incidence rates are reported in high-income and well-developed regions, including Europe, Australia, North America, and Eastern Asia, suggesting a link with lifestyle and dietary factors [8,9,10]. Having an impact on gut microbiota, alcohol consumption, and dietary choices constitute the most important modifiable risk factors for CRC [11,12,13].

The gut microbiota plays a crucial role in maintaining intestinal homeostasis through numerous functions, including the breakdown of non-digestible dietary components, the renewal of epithelial cells, the maintenance of intestinal mucosal integrity, immune system modulation, and the secretion of antimicrobial substances. It also protects against infections by competing with pathogenic organisms for nutrients and receptors while promoting overall health through the synthesis of vitamins, regulation of fat reserves, and energy storage [14]. The state of eubiosis, which refers to the balance between the host and its microbiota, is essential for proper gut function and overall health [15]. Gut microbiota is highly sensitive to environmental factors such as dietary macro- and micronutrients, infections, and lifestyle, which can lead to alterations in its composition and function, a condition known as dysbiosis [16,17]. Gut dysbiosis is often associated with intestinal inflammation, which may evolve into IBD and CRC [18]. Specifically, a reduction in the abundance of beneficial bacteria or an overgrowth of pathogenic organisms such as *Bacteroides*, *Prevotella*, *Proteobacteria*, *Eubacterium*, *Fusobacterium*, *Proteobacteria*, *Escherichia coli*, *Clostriudium*, and *Salmonella* spp. can disrupt the balance of microbiota, creating an environment that promotes inflammation and tumorigenesis [19,20].

Recently, prebiotics, probiotics, and postbiotics capable of restoring eubiosis have emerged as potent dietary supplements for the prevention and post-surgery adjuvant therapy of CRC [21]. The most important metabolites released by the intestinal microbiota with beneficial effects on human health are short-chain fatty acids (SCFAs), including acetate, butyrate, and propionate. These postbiotics are produced during the fermentation of non-digestible plant fibers (prebiotics) in the diet. Butyrate is by far the most abundant one and serves as the primary energy source for intestinal cells [22]. Butyrate has a wide range of beneficial effects on health. By interacting with its receptors, it modulates immune system activity, promotes the release of anti-inflammatory cytokines, and exhibits various anti-tumor properties, including inducing autophagy and apoptosis and reducing the proliferation of neoplastic cells. Additionally, butyrate plays a crucial role in reducing oxidative stress and reinforcing the intestinal barrier, preserving gut integrity [23,24].

This narrative review presents the most recent knowledge on the role of autophagy and inflammation in CRC and how probiotics and their metabolites modulate these processes to prevent and cure CRC. To prepare this review, we searched for scientific publications in public databases using appropriate combinations of keywords, including probiotics, postbiotics, microbiota, microbiome, colorectal cancer, inflammation, autophagy, immunity, engineered microorganisms, etc. The relevant literature was selected according to four main criteria: 1. reputation of the journal and expertise of the authors; 2. quality of methodology, description of mechanisms, and translational significance of the results; 3. discussion of controversial issues where applicable; 4. most recent publications were given priority.

### 1.2. Microbiota and Colorectal Cancerogenesis: The Role of Genetics and Epigenetics

Most cases of CRC are sporadic and develop from polyps through the adenoma–carcinoma sequence following a characteristic sequential accumulation of genetic and epigenetic mutations in well-defined oncogenes, tumor suppressor genes, and DNA repair genes in 10 years or more [25,26]. CRC hereditary predisposition is linked to germline inherited monoallelic mutations in tumor suppressor or DNA repair genes [2,27]. Inactivation (by mutation or epigenetic silencing) of the second allele creates the conditions for the initiation step of tumorigenesis, which can be followed by additional mutations or epimutations in oncogenes and tumor suppressor genes that eventually lead to frank carcinoma.

Around 80% of CRCs are associated with mutations in the Wnt/β-Catenin signaling pathway [28], which is involved in key processes such as adult tissue renewal and homeostasis, hematopoietic stem cell maintenance, and regulation of cell proliferation, migration, and differentiation [29]. Loss-of-function mutations in the oncosuppressor *APC*, mutations in *CTNNB1* (β-Catenin gene), and the excessive presence of Wnt ligands in the tumor microenvironment (TME) disrupt these regulatory mechanisms, resulting in uncontrolled tumor growth [30,31]. The *APC* mutation specifically initiates the formation of adenomas or intestinal polyps, some of which progress to adenocarcinomas due to the accumulation of additional mutations, including those in *KRAS*, *DCC*, *TP53*, and *SMAD4* [32].

Genetic and epigenetic mutations in genes relevant to colorectal carcinogenesis are influenced by and interact with environmental factors, such as diet, lifestyle, therapy, and gut microbiota, thereby accelerating the development and progression of the disease [33]. The following findings exemplify how gut microbes can influence gene function and, consequently, the development of colorectal cancer.

Genotoxic pks+ *Escherichia coli* strain and *Bacteroides fragilis* have been shown to cause DNA mutations through the production of free radicals and of genotoxins (namely, colibactin), thus contributing to oncogenic activation and CRC development [34,35]. Similarly, *Enterococcus faecalis* has been shown to cause chromosomal instability and aneuploidy in enterocytes through oxidative stress [36].

Dysbiosis may contribute to CRC development also via epigenome changes [37]. For instance, antibiotic therapy-induced and diet-dependent depletion of SCFA-producing bacteria in the intestine may influence chromatin accessibility and transcription in a way that promotes CRC progression [38,39,40,41]. Similarly, genotoxins produced by *Bacteroides fragilis* have been shown to modulate the methylation and chromatin accessibility of genes involved in CRC development [42]. The microbe composition also influences the intestine epithelial cell expression of long non-coding RNAs and of microRNAs that have an impact on intestine homeostasis, although their mechanistic role in CRC development and progression remains to be elucidated [43].

On the contrary, eubiosis can prevent mutagenic and epimutagenic events, thus protecting from CRC development. In vitro studies have shown that certain *Lactobacillus* strains possess anti-genotoxic and anti-mutagenic properties, effectively reducing mutagen levels and thereby lowering the risk of CRC initiation [44,45]. In a mice model of colorectal carcinogenesis, the supplementation with probiotic strains of *Bifidobacterium bifidum* and *Lactobacillus acidophilus* reduced the expression of the oncomiRNAs miR-135b and miR-155 while increasing the expression of miR-26b and miR-18a, and these changes were associated with downregulation of the oncogene K-Ras and the upregulation of the tumor suppressors APC and PTEN [46]. In this same line, the supplementation with *Bifidiobacterium longum* inhibited colorectal carcinogenesis in mice via downregulation of miR-155 and miR-21a and up-regulation of the tumor suppressive miRNAs miR-145 and miR-15a [47].

Finally, the inhibition of cell proliferation and induction of cell death in CRC cells by butyrate has been (at least partly) attributed to its capability to modulate the expression of pro-apoptotic and cell cycle genes via HDAC inhibition [48].

### 1.3. Colorectal Cancerogenesis: The Role of Autophagy

Autophagy is a catabolic process highly conserved in eukaryotic cells that maintains cell homeostasis through the lysosome-mediated degradation of damaged (unfolded, oxidized), aged, or redundant cellular components, as well as the destruction of intracellular pathogens. Based on the mechanism that directs the degrading material (cargo) to the lysosome, autophagy is classified into three types: (i) macroautophagy (where the cargo is sequestered within the autophagosomes), (ii) microautophagy (small portion of the cytoplasm is internalized by invagination of the lysosome membrane), and (iii) chaperon-mediated autophagy (internalization of selected proteins is mediated by HSC73) [49]. A more in-depth description of the autophagy machinery and its biochemical regulation by extracellular signals and by genetics and epigenetics can be found in excellent reviews [49,50,51]. Here, we will only give a glance at macroautophagy (simply referred to as autophagy) since this pathway plays a major role in macromolecular degradation and turnover. This process is characterized by the sequestration of the cargo within autophagosomes (double-membrane organelles) that subsequently fuse with endosomes and lysosomes, forming the autolysosomes in which the cargo is fully degraded, and the substrates are exported in the cytosol for reutilization [40]. Autophagy is repressed by growth signals and an abundance of nutrients (glucose, amino acids) through the activation of mTORC1, while it is induced in conditions of nutrient depletion and reduced oxidative phosphorylation through the activation of AMPK [49]. Autophagy is a pro-survival pathway that opposes apoptosis, yet when hyper-induced because of overwhelming cellular toxicity it can precipitate autophagic cell death [49]. This is reflected in cancer, where autophagy may play two opposite roles: (i) as anti-cancer, it can prevent carcinogenesis by eliminating damaged subcellular structure and protecting the genome by contrasting cell mass growth and inhibiting cell proliferation and cell migration and by promoting cancer cell dormancy, whereas (ii) as pro-cancer, it can favor survival in floating metastatic cells and can protect the cancer cell from chemo- and radio-induced damages [52]. Autophagy also plays a role in reshaping the TME by reducing angiogenesis and inflammation, relieving immune suppression, favoring tumor dormancy, and promoting the reversal of the phenoconversion of cancer-associated fibroblasts (CAFs) into normal fibroblasts [53,54,55]. Stimulation of autophagy also re-educates M2 tumor-associated macrophages [56].

The involvement of autophagy in CRC is suggested by the finding of microsatellite instability and frameshift mutations in the autophagy-related genes *ATG2B*, *ATG5*, and *ATG9B* [57]. The role of autophagy in colorectal carcinogenesis is multifaceted and apparently controversial, as it is likely stage- and context-dependent [58,59]. For instance, in a small cohort of CRC patients, it was found that the expression of *ATG10* was positively associated with metastasis and poor prognosis [60], yet another study proved that downregulation of *ATG10* in CRC was associated with EMT and liver metastasization [61]. Upregulation of autophagy genes or proteins in cancer cells could reflect a stress response rather than the cause of malignancy. For instance, knock-down of autophagy was reported to improve 5-FU chemotherapy toxicity in CRC [62].

Particularly when assessing the expression of autophagy genes or proteins in pathological specimens, it is important to also consider the tumor microenvironment since autophagy is differentially dysregulated in the cell depending on the surrounding conditions. For instance, in the hypoxic niche, autophagy is induced to promote the survival and proliferation of colorectal cancer stem cells [63]. Another important caveat in assigning a role for autophagy in cancer is related to the methods used to assess it. Older studies using chloroquine (which alkalinizes lysosomal pH) or 3-methyladenine (a PI3K inhibitor) should be treated with caution because of their nonspecific effects. The role of autophagy in cancer is better appreciated in models where it is genetically manipulated, although we cannot ignore the possibility of off-target effects, especially when using miRNAs. In *Apc*^+/−^ mice genetically predisposed to CRC, the conditional ATG7 knock-down in enterocytes prevented CRC development by improving T-cytotoxic immune response and was associated with altered microbiota [64]. However, in this same *Apc*^+/−^ CRC model, the heterozygous deletion of ATG5 led to an increased tumor burden [65]. Additionally, autophagy could protect *Apc*^+/−^ mice from *E. coli* colibactin-induced colorectal carcinogenesis [66].

In several in vitro and in vivo CRC models, the post-transcriptional silencing of autophagy-related genes was found to inhibit chemoresistance, cancer growth and invasiveness, and metastasis [67,68,69].

**Figure 1:**
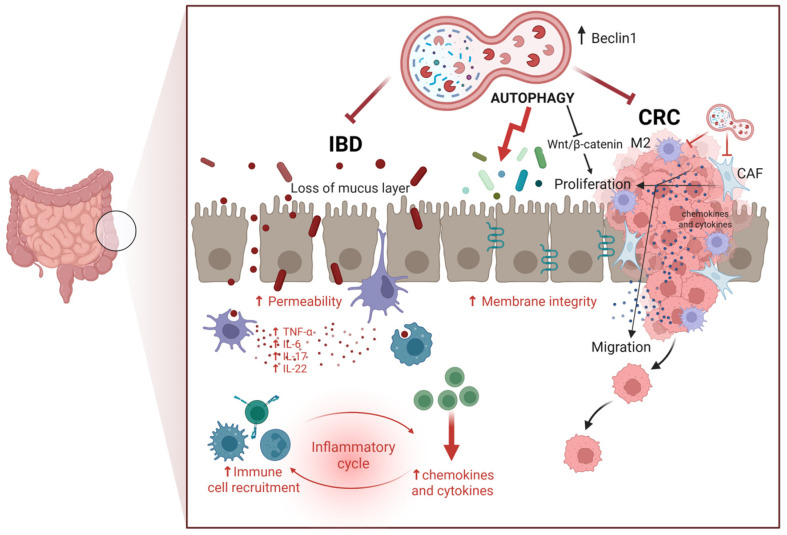
Role of autophagy in regulating intestinal homeostasis. Autophagy counteracts colorectal cancer (CRC) by limiting cell proliferation and migration, mainly through inhibition of the Wnt/β-Catenin pathway) while also mitigating inflammation associated with inflammatory bowel disease (IBD), a condition that can progress to CRC. Autophagy in epithelial cells is also important for maintaining cell-to-cell adhesion and producing mucus, which prevents colonization by pathogenic bacteria. These activities highlight the dual protective role of autophagy in maintaining intestinal integrity and preventing tumorigenesis (Created in BioRender. Isidoro, C. (2025) https://BioRender.com/pzq0dhg).

On the other hand, there is evidence that supports the anti-cancer role of autophagy in inhibiting CRC growth, progression, and metastasis [70,71]. For instance, pharmacologic inhibition of mTORC1 and mTORC2 suppressed CRC growth in an autophagy-dependent manner [72]. Consistent with the latter, increased expression of the autophagy-related tumor suppressor gene *BECLIN-1* is associated with better overall survival in CRC patients undergoing chemotherapy [73]. In several in vitro and in vivo CRC settings, the miRNA-mediated knock-down of autophagy was associated with increased resistance to therapy, tumor growth, invasiveness, metastasization, and poor prognosis, such as was the case with MiR-338-5p silencing of PI3KC3 [74] and miR-183-5p silencing of ATG5 [75]. Further, autophagy stimulates the immune system functions, improves pathogen clearance, and downmodulates the production of inflammatory cytokines, thus preventing the onset of intestinal inflammatory diseases that could evolve in CRC [76,77,78,79]. Connected with this, autophagy has been shown to improve the intestinal barrier, enhancing the cell-to-cell tight junction [80,81]. To be noted, TNFα induces intestinal permeability through inhibition of autophagy [82]. Figure 1 schematizes the beneficial roles of autophagy in preventing CRC.

As a final note, in transgenic mice knock-down for ATG5 in enterocytes, the intestinal microbiota was dramatically changed, with a prevalence of bacteria promoting inflammatory diseases [83].

### 1.4. Colorectal Cancerogenesis: The Role of Inflammation and Its Interplay with Immune Cells

Chronic inflammation is a crucial hallmark of cancers [84]. Inflammatory immune cells and the associated cytokines in the TME are thought to play a double-faced role in the initiation and progression of CRC [85]. Inflammation starts as a defensive response of the immune system to pathogens or tissue damage. In response to intestine-localized inflammation, cytokines produced by gut immune cells (TNF-α, IL-1β, and IL-6) stimulate the crosstalk among the gut microenvironment cells [85]. However, the persistent activation of the inflammatory response leads to an excess release of cytokines, chemokines, and growth factors that eventually contribute to malignant transformation, cancer growth, and progression [86]. The sequential phases of induction of inflammation, stimulation of proliferation, and tumorigenesis following microbial dysbiosis and metabolic alterations have been clearly elucidated in animal models of colitis-associated colorectal cancer (CAC) [87].

Inflammatory bowel disease (IBD) is associated with oxidative stress and DNA damage, resulting in genetic alterations that cause dysplasia and stimulate the proliferation of epithelial cells, eventually leading to tumor development [88]. Accordingly, IBD patients have approximately a two- to three-fold increased risk of developing CRC compared to the general population [89,90].

In CRC, a complex interplay exists between intestinal microbes, epithelial cancer cells, and stromal cells (including CAFs, tumor-associated macrophages (TAMs), and immune cells) that dynamically change their composition and phenotype (and behavior) within the TME according to the availability of nutrients, growth factors, cytokines, and probiotic metabolites. The latter alters the cellular composition of the TME by recruiting and reprogramming stromal cells, and this reflects in a dynamic change from suppressive to permissive TME in the different areas of the tumor, which, in turn, will determine the behavior of CRC cells in terms of growth, metastasization, dormancy, or chemoresistance. Reciprocally, cancer cells and stromal cells release a plethora of inflammatory cytokines that ultimately affect the microbiota, further contributing to the creation of a permissive TME for the growth and metastasization of CRC [91]. IL-6 is a master inflammatory cytokine, along with TNF-α, IL-17, and IL-22, promoting the development and progression of CRC [92,93]. IL-6 secreted by CRC-associated fibroblasts stimulates angiogenesis, inhibits CRC cell death, and induces CRC cell proliferation and motility [94,95]. Further, IL-6 induces the release of TGF-β, thus promoting EMT (Epithelial-to-Mesenchymal Transition), activates the proliferation pathways (such as ERK/MAPK, PI3K, and Wnt/β-Catenin), promotes M2 TAM polarization, and suppresses the immune response [95,96]. The serum levels of IL-6 increase during the progression from colorectal adenoma to carcinoma, and, consistently, CAC and CRC patients with high serum levels of IL-6 present with tumors of large size, relapse, and shorter overall survival [97]. Macrophages are key players in the TME, where they can dynamically change their phenotype from M1-like to M2-like depending on the extracellular stimuli. M1-like macrophages, identified by the expression of CD80 and CD86, show glycolytic metabolism and dialogue with T_H_1 and exert a pro-inflammatory and anti-tumor function, while M2-like macrophages (and their subtypes), identified by the expression of CD206 and CD163, show phosphorylation oxidative metabolism and dialogue with T_H_2 and exert an anti-inflammatory, immune-suppressive, and pro-tumorigenic function [98,99]. Macrophages reprogram their metabolism and phenotype in response to the extracellular signals. In the tumor niche, the crosstalk between cancer cells and macrophages determines the fate of CRC. Mimicking this situation in vitro, CRC cells co-cultured with CD68, CD204, and CD206-positive M2-like macrophages showed an increased rate of proliferation and colony formation [56]. Interestingly, this effect was abrogated when the M2-like macrophages were pre-exposed to rapamycin to induce autophagy, highlighting the strict interplay between autophagy and inflammation in colorectal carcinogenesis [56]. As stated above, another layer of complexity is due to the ability of microbiota and its metabolites to modulate the phenotype of macrophages within the TME [100,101]. In the gut of CRC patients, *Bacteroides* and *Bacillus faecalis* are associated with an increased infiltration of Tregs and consequent M2-TAM accumulation in the TME [102]. Indeed, in a colitis murine model, propionate and butyrate were shown to promote the accumulation of anti-inflammatory M2-like macrophages through the induction of Treg [103]. Summing up, M1-mediated inflammation starts as a first line of defense against colorectal cancer, stimulating T_H_1-mediated immune response, yet with time, cancer cells secrete cytokines that reprogram the macrophages into M2-type that are anti-inflammatory but, at the same time, immune-suppressive and supportive of tumor growth. These M2-TAMs also recruit fibroblasts that, in the TME, become CAF-releasing IL-6 and other inflammatory cytokines. This scenario resembles that of a wound that never heals, as brilliantly defined by Harold F. Dvorak in 1986 [104]. In this context, probiotics and their metabolites may promote “healing” by turning off excess inflammation in the intestinal TME (see Section 1.6).

### 1.5. Dysbiosis and Colorectal Cancerogenesis: The Role of Diet and Microbiota

CRC incidence is strongly associated with dietary habits [105]. The Western diet, characterized by a high intake of red and processed meats, saturated fats, alcohol, and sugar, is associated with an increased risk of CRC [13,106]. This is linked to the oxidative metabolism of foods that typically make up the Western diet [107]. A fat-rich diet associates with inflammatory adipose tissue and contributes to colorectal carcinogenesis [108]. In contrast, diets rich in low-fat dairy products such as fruits, vegetables, legumes, fiber, whole grains, and fish, combined with moderate alcohol intake, have been shown to have a protective effect against CRC. This protective effect is attributed to their content in prebiotics and specific micronutrients, such as calcium and magnesium, vitamins like vitamin D and vitamin B6, and polyphenols [12].

The gut microbiota is a dynamic community of several types of microbes, including archaea, eukaryotes, bacteria, viruses, and parasites, that have evolved to grow and survive in the gastrointestinal tract. Within the microbial community, bacteria are the most abundant group, comprising approximately 100 trillion individuals [109]. These bacteria belong to several phyla, including *Firmicutes*, *Bacteroidetes*, *Actinobacteria*, *Fusobacteria*, *Proteobacteria*, *Verrucomicrobia*, and *Cyanobacteria*, with *Firmicutes* and *Bacteroidetes* constituting 90% of the total bacterial population [110]. Throughout life, the composition of the gut microbiota changes since it is influenced by age, sex, and lifestyle factors such as physical exercise, sedentary behavior, smoking, diet, and the use of antibiotics, creating a unique microbial profile for each individual [16,17,19]. Interestingly, all these factors impact autophagy modulation, and it has been shown that impaired autophagy in intestinal cells alters the microbiome composition [83].

The microbiome of CRC patients is altered, with a reduction in *Bacteroides, Firmicutes*, and *Lactobacillus,* along with a remarkable increase in *Fusobacterium* species, among others [111,112]. This imbalance results in mucosal permeability, triggers inflammation, and promotes the activation of the Wnt/β-Catenin mitogenic pathway.

The bacteria in the gastrointestinal tract can ferment non-digestible carbohydrates, generating SCFAs. The main SCFAs are acetate, propionate, and butyrate. SCFAs exhibit various beneficial effects, such as maintaining intestinal barrier integrity, regulating immune function, providing protection against pathogens, and promoting anti-inflammatory responses [76,113]. SCFAs also display anti-tumor properties by inducing apoptosis, reducing the proliferation of neoplastic cells, and mitigating oxidative stress [114]. The homeostatic effects of SCFAs on the colonic epithelium are mediated by SCFA-specific transporters. Specifically, MCT1 is located on both the apical and basolateral membranes, MCT4 is found on the basolateral membrane, and SMCT1 and SMCT2 are located on the apical region of intestinal cells [115].

Dysbiosis is a common feature of intestinal diseases, including IBD, CAC, and CRC [116]. Consistently, patients with CRC exhibit low microbial biodiversity, with specific bacteria, such as *Fusobacterium nucleatum*, *Escherichia coli*, *Enterococcus faecalis*, *Streptococcus gallolyticus*, and *Bacteroides fragilis* present in higher abundance in their gut [20,117,118].

Below, we detail the mechanisms through which the main pathogenic bacteria may promote colorectal cancerogenesis.

*Fusobacterium nucleatum* invades the intestinal epithelium through the expression of the adhesion protein FadA. This protein forms a complex with E-cadherin, increasing intestinal barrier permeability [101] and enhancing β-Catenin activity [119], which promotes cancer cell growth. Additionally, *F. nucleatum* suppresses immune cell activity by binding specific inhibitory receptors on natural killer (NK) cells and T cells [103]. An increase in tumor-promoting cytokines, including IL-17 and TNF-α, in CRC is associated with the activation of the NF-κB pathway by *F. nucleatum* [120].

A similar role is attributed to *Bacteroides fragilis*, which produces a metalloprotease that cleaves E-cadherin, disrupting the intestinal barrier [121]. It also induces the nuclear translocation of β-Catenin [122] and stimulates the production of IL-8 and IL-17, triggering an aberrant immune response and promoting tumorigenesis [123].

Additionally, *Salmonella*, *Clostridium difficile*, and *Prevotella* are also linked to CRC pathogenesis through the activation of the β-Catenin [124,125].

*Streptococcus gallolyticus* drives CRC carcinogenesis by inducing inflammation through IL-1, COX-2, and IL-8 pathways [115], as well as by increasing the levels of β-Catenin and C-MYC [126].

**Figure 2:**
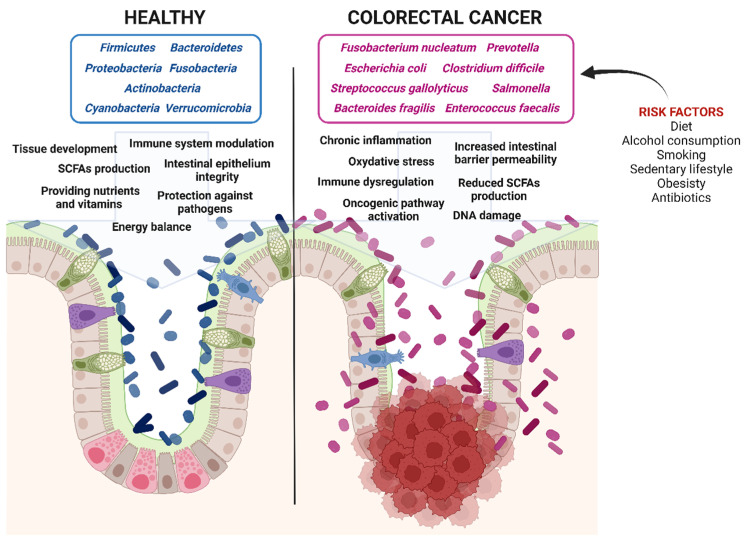
Impact of gut microbiota on colorectal cancer progression. Healthy gut microbiota maintains epithelial integrity and energy balance, prevents inflammation, provides essential nutrients and vitamins, produces short-chain fatty acids (SCFAs), and protects against pathogen invasion. In contrast, dysbiotic gut microbiota increases intestinal permeability, promotes chronic inflammation and oxidative stress, induces DNA damage, activates oncogenic pathways, and reduces SCFA production, all of which contribute to colorectal cancer development (Created in BioRender. Isidoro, C. (2025) https://BioRender.com/pzq0dhg).

The intestinal dysbiosis is also accompanied by a reduction in butyrate-producing bacteria, such as *Eubacterium*, *Roseburia*, and *Faecalibacterium*, further creating a microenvironment favorable to CRC development and progression [127] (Figure 2).

### 1.6. Beneficial Effects of Probiotics and Postbiotics in Colorectal Cancer: Impact on Inflammation and Autophagy

Probiotics are living bacteria that elicit beneficial health effects by restoring eubiosis. Probiotics and their metabolites (postbiotics) exhibit anti-cancer properties through various mechanisms, such as altering gut microbiota composition, reducing intestinal pH levels, neutralizing mutagens or carcinogens, enhancing immune responses while dampening inflammation, and promoting autophagy, apoptosis, and cell differentiation processes [128,129,130]. Further, probiotics enhance the activity of antioxidant enzymes such as superoxide dismutase, catalase, and glutathione peroxidase, which counteract oxidative stress and provide additional protection against CRC development [130]. Here, we will emphasize the impact on the immune–inflammation response and autophagy.

One of the ways probiotics combat inflammation is through the interaction of bacterial products with Toll-like receptors (TLRs) expressed on dendritic cells, CD4+ T cells, and macrophages. TLR2 (that engages with peptidoglycan from Gram-positive bacteria), TLR4 (that engages with Lipopolysaccharide from Gram-negative bacteria), and TLR5 (that engages with flagellin) signal to immune cells and discriminate the presence of commensal or pathogenic bacteria that should be tolerated or rejected, respectively [131]. Excess stimulation of TLR4 versus TLR2 has been associated with a higher risk of CRC [132,133]. For instance, through these interactions, *Lactobacillus rhamnosus* and *Lactobacillus reuteri* trigger the differentiation of naïve CD4+ T cells into Treg cells, which produce IL-10 and reduce the production of TNF-α [134]. Polysaccharide A from *Bacteroides fragilis* could dampen inflammation (while inducing tolerance) through direct interaction with TLR2 on Treg (not on dendritic cells) to induce the secretion of IL-10 that, in turn, suppressed the pro-inflammatory T17 [135]. Furthermore, probiotic strains of *Lactobacillus acidophilus*, *helveticus*, and *plantarum* inhibited the activation of NF-κB by interacting with TLR4 on macrophages, thereby reducing the production of pro-inflammatory cytokines such as TNF-α, IL-1β, and IL-6 [136]. Additionally, probiotics promote the differentiation of B cells into IgA-producing plasma cells, enhancing mucus production and preventing the adhesion of microorganisms to intestinal epithelial cells, thereby reducing the risk of pathogen penetration [130,137,138]. Another beneficial anti-cancer effect of certain probiotics is the induction of autophagy [128]. The expression of the autophagy genes *ATG7*, *ATG5*, and *ATG16* increased in CRC cells incubated with a mixture of sonicated *Bifidobacterium bifidum, longum*, and *infantis* [139]. Soluble metabolites of *Lactiplantibacillus plantarum OC01* were found to reduce cell proliferation and migration of CRC cells cultivated as 2D- or 3D-spheroids, even in the presence of the inflammatory cytokine IL-6, an effect linked to induction of autophagy and β-Catenin degradation [140]. Additionally, these metabolites changed the secretome of CRC cells, reducing the release of cytokines (particularly TGF-β) so that tumor-promoting M2-like macrophages were reverted into an anti-tumor M1-like phenotype [141].

**Figure 3:**
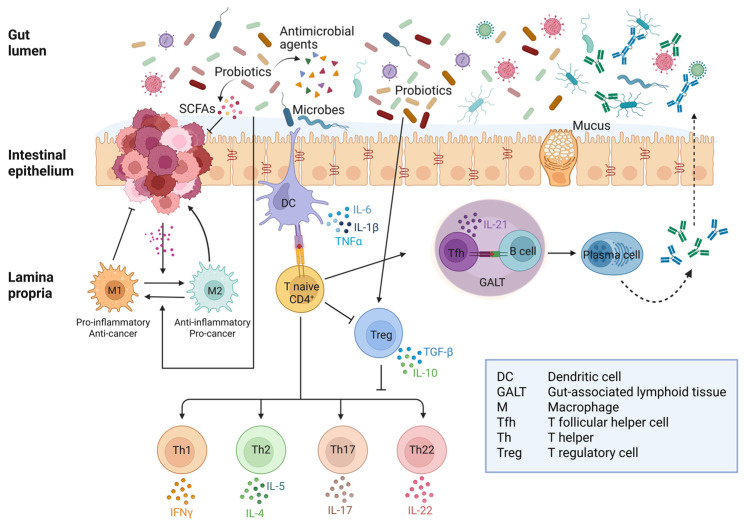
Crosstalk between immune and inflammatory responses to gut microbiota in healthy intestine and colorectal cancer. In the presence of pathogenic bacteria, dendritic cells trigger an immune response through Th cells while inhibiting Treg differentiation. On the other hand, commensal bacteria are tolerated by promoting Treg and suppressing Th response. Colorectal cancer cells release soluble factors that promote the phenoconversion of macrophages into M2-type TAMs, which release factors that support tumor growth. Probiotics and their postbiotics downregulate the inflammatory response by activating Treg cells, which then suppress Th17 and Th22. Moreover, SCFAs can revert M2-type into M1-type TAMs with anti-tumor activity (Created in BioRender. Ferraresi, A. (2025) https://BioRender.com/5tjh823).

Figure 3 illustrates (in a simplified manner) how probiotics and postbiotics influence the immune–inflammatory response and the crosstalk between different types of immune cells. A more detailed description of the mechanistic interaction between probiotics and intestinal immune cells can be found in [138,142], and a more in-depth discussion of the anti-inflammatory mechanisms of probiotics and postbiotics in the gut can be found in [76,138]. It is to be stressed that the crosstalk between immune cells in the lamina propria differs in healthy, inflammatory diseases and colorectal cancer. As an additional layer of complexity, the interplay between the microbiota (and probiotics with their postbiotics) and the cancer cells change with time dynamically and spatially, thus leading to a continuous rearrangement of the cellular composition in the stroma.

In this context, the picture is further complicated by the fact that autophagy in epithelial intestinal cells influences the microbiota, limiting the growth of pathogenic bacteria that induce inflammation and intestinal permeability [83]. However, autophagy is dysregulated and differentially modulated in CRC cells, depending on the availability of oxygen, nutrients, growth factors, and cytokines, which implies different scenarios in different niches.

The anti-carcinogenic effects of probiotics are also attributed to the production of SCFAs, which can act on both epithelial and immune cells [143]. Butyrate is the primary SCFA absorbed by the intestinal epithelium and serves as the main energy source for colonocytes, while acetate and propionate mainly reach the liver [22]. Butyrate promotes the integrity of the gut barrier by regulating the genes that encode for the tight junction proteins, such as claudin-1, zonula occludens-1, and occludin [144]. Furthermore, by enhancing the expression of Mucin 2, butyrate reinforces the mucus layer of the gut epithelium, which is essential for protection against pathogens and immune response regulation [145]. SCFAs, particularly butyrate, negatively regulate the inflammatory signaling pathway mediated by the inflammasome complex NLRP3, thereby inhibiting macrophage activation [56]. In M2-type macrophages, butyrate promotes the production of the anti-inflammatory cytokine IL-10 while reducing the production of inflammatory cytokines such as TNF-α, MCP-1, and IL-6 through the activation of GPR43 (G-protein coupled receptor 43) [146].

Butyrate has been shown to prevent the activation of CD4+ T cells isolated from normal gut *lamina propria* through inhibition of HDAC in a dose-dependent manner, and this decreased the formation and the cytokine production of Th1, Th17, and Th22 cells [147].

The beneficial effects of SCFAs extend to the epithelial cells. As mentioned above, dysregulation of autophagy in intestinal epithelium may lead to impaired bacteria clearance and pathogen invasion and overall gut microbiota dysbiosis and exacerbated chronic inflammation that ultimately increases the risk of CRC [76,83]. Butyrate was shown to stimulate autophagy in CRC cells through the activation of the LKB1-AMPK pathway [148]. Of relevance, in colorectal cancer cells, there is a crucial interplay between the Wnt pathway and autophagy. Activation of the Wnt/β-Catenin signaling pathway has been shown to inhibit autophagy while promoting CRC growth [149]. Butyrate was reported to downregulate the Wnt/β-Catenin pathway, thus counteracting the early stages of CRC development. In resting epithelial cells, β-Catenin is bound to E-Cadherin and to the APC destruction complex that directs its ubiquitination and proteasomal degradation so that its accumulation in the cell and translocation in the nucleus is prevented. Proteolytic degradation of E-Cadherin by metalloproteases secreted by *Bacteroides fragilis* frees β-Catenin [122]. Further, most CRCs have mutations in APC and/or CTNB1 that impair the degradation of β-Catenin [150,151]. By inducing autophagy as an alternative mechanism to the proteasomal degradation, which is ineffective in *APC-* and *β-Catenin*-mutated CRC, butyrate promoted the autophagic sequestration and degradation of β-Catenin, and this inhibited the proliferation and migration of CRC cells [152].

**Figure 4:**
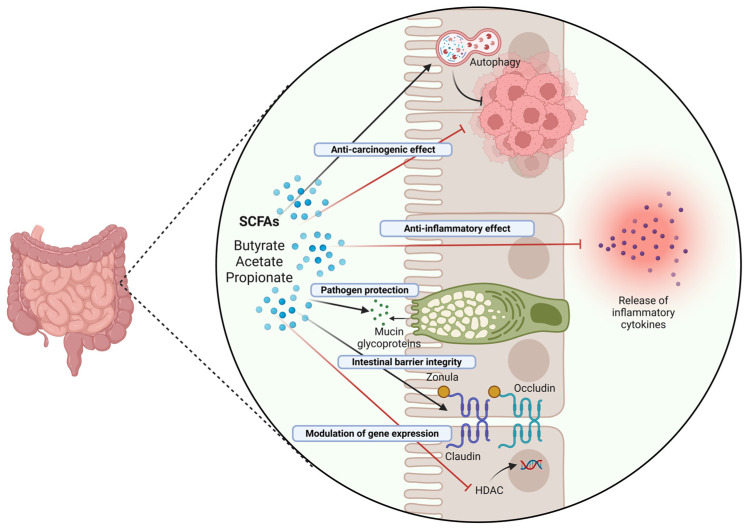
Role and mechanisms of action of SCFAs in the intestinal epithelium. SCFAs, particularly butyrate, exhibit anti-carcinogenic effects in the intestine through a range of mechanisms, including induction of protective autophagy (and correlated downregulation of the Wnt/β-Catenin pathway), immunomodulation of the inflammatory response, epigenetic modulation of gene expression, and stabilization of intestinal barrier integrity (Created in BioRender. Isidoro, C. (2025) https://BioRender.com/pzq0dhg).

Figure 4 schematizes the mechanisms of protective action of butyrate on the intestinal mucosa.

### 1.7. Challenges in Probiotic-Based Therapy and Future Directions

The previous paragraphs have illustrated how probiotics and postbiotics help reshape gut microbiota by promoting the growth of beneficial bacteria, improving the inflammatory and immune responses, and stimulating protective autophagy, overall reducing the risk of CRC development. Several preclinical and clinical evidence support the preventive, therapeutic, and adjuvant perioperative efficacy of probiotics and their soluble metabolites in CRC [153,154].

Below are some examples to give an idea of the preventive and therapeutic potential of this approach. In animal models, the treatment with probiotics of the *Lactobacillus* genus decreased the formation of tumors, reduced the growth of aerobic bacteria in the intestine, and restored the correct host immune functions [155,156,157]. A mixture of *Lactobacillus* species (namely two strains of *L. plantarum* and one strain each of *L. reuteri*, *L. brevis*, and *L. rhamnosus*) was found to inhibit the in vitro proliferation of human CRC cell lines and the in vivo growth of CRC in mice through modulation of the expression of APC (which was increased) and of β-Catenin (which was decreased) [158]. In CAC models, the administration of VSL#3, a probiotic mixture containing *Bifidobacterium breve*, *B. infantis*, *B. longum*, *Lactobacillus acidophilus*, *L. bulgaricus*, *L. casei*, *L. plantarum*, and *Streptococcus thermophilus*, reduced the incidence of high-grade dysplasia and prevented the development of CRC. These probiotic strains contrasted colorectal carcinogenesis in mouse models of Dextran Sulfate Sodium (DSS)- and Azoxymethane (AOM)-induced colitis, reducing the activation of STAT3 and the expression of BCL-2 and IL-6 [159,160].

In patients with CRC, there is a marked reduction in butyrate levels, a condition predisposing to intestinal permeability, increased inflammation, and dysbiosis [161,162]. The consumption of probiotics such as *Lactobacillus* and *Bifidobacterium* has been shown to increase the production of butyrate in the colon. Consistently, the supplementation with *L. casei*, *L. acidophilus*, *L. lactis*, *B. bifidum*, *B. longum*, and *B. infantis* improved the quality of life for CRC patients by ameliorating the inflammatory status in the intestine [163]. In CRC patients, probiotics were shown to synergize with chemotherapy by improving their therapeutic efficacy and reducing the side effects. The co-administration of *L. acidophilus* and *L. casei* improved the efficacy of the chemotherapeutic drug 5-FU, increasing its pro-apoptotic efficiency [164]. In this same line, CRC patients co-treated with *L. rhamnosus* could be treated with lower doses of 5-FU and showed reduced episodes of diarrhea and abdominal pain and required shorter hospitalization compared to patients treated with only the chemotherapeutics [165]. Another study proved a synergism between 5-FU and the cell-free supernatant of *Lactobacillus plantarum* [166]. Probiotic treatment showed efficacy as perioperative therapy as well. *Lactobacillus* bacteria administered before and after surgery reduced proliferation, growth, and invasion of CRC cells, decreased enteropathogenic bacteria in blood, improved diarrhea, restored the integrity of gut mucosa, and stimulated the systemic immune system [167,168]. Post-operative complications were reduced in CRC patients treated with *Bifidobacterium* and *Lactobacillus* [169,170]. Patients treated with chemotherapy supplemented with *Bifidobacterium breve* demonstrated increased microbiota diversity, a lower frequency of fever, and a reduced risk of infections [169]. The administration of specific *Lactobacillus* and *Bifidobacterium* strains reduced radiotherapy-related gut toxicity and diarrhea [171,172].

Probiotics were revealed to be useful for CRC treatment also in combination with immunotherapy. In this respect, it was found that SCFA-producing bacteria were more abundant in responders, while *Bacteroides* were more abundant in non-responders to PD-1/PD-L1 therapies [173]. Particularly, in intestinal cancer patients, an increased ratio of *Prevotella* and *Ruminococcaceae* vs. *Bacteroides* was associated with a good response to immune checkpoint inhibitor therapy [174].

To give a balanced overview of the state-of-the-art, we must also consider the other side of the coin. Two of the most important issues that need to be taken into account are that probiotic treatment may not work in immunocompromised patients and that it is not advisable for patients with serious dysbiosis associated with a leaky intestinal barrier. In the latter case, the risk of septicemia and acute complications must be carefully considered. Here, the use of postbiotics (which do not contain living bacteria), enriched with enzymes, proteins, vitamins, and short-chain fatty acids, could be an alternative to bring the benefits at no or very minimized risk.

In Table 1, we summarize the main preclinical studies and clinical trials with their pros and cons and limitations.

To maximize the benefits of probiotic and postbiotic treatments, we need to make use of the “omics” technologies to better understand the potential of probiotic strains. One other limitation is that most studies are focused on a relatively small number of species, many of which are not indigenous to the human and mouse gut.

The future is to personalize probiotic therapy for patients based on the accurate assessment of the type and severity of intestinal inflammation, immune performance, genetic and epigenetic predisposition, microbiota composition, and lifestyle factors. To this end, we should take advantage of testing probiotics and postbiotics efficacy (and dose) in appropriate in vitro models using organoids (that include intestinal and immune cells), the so-called “gut-on-chip”, and even better if reconstructed in a microfluidic chamber mimicking the dynamic changes in the TME [198,199].

## 2. Highlights and Concluding Remarks

Dysbiosis plays a significant role in the development and progression of CRC. Environmental factors, including diet, infections, and drugs, can disrupt the intestinal bacterial flora, which consequently may affect SCFAs and vitamin synthesis, increase stress responses, cause immune dysregulation, and increase susceptibility to DNA alterations. Over time, these changes lead to chronic inflammation, immune dysfunction, and metabolic alterations that can contribute to the development of conditions such as allergies, obesity, irritable bowel syndrome, IBD, and eventually CRC.

Autophagy plays a crucial role in maintaining cellular homeostasis through the turnover of damaged proteins and organelles. Gut bacteria and their secretions influence the level of autophagy in intestinal epithelial cells, and reciprocally, dysregulation of autophagy can influence the composition and function of the gut microbiota. This bidirectional relationship, in which autophagy impacts microbial balance and dysbiosis affects autophagic regulation, plays a significant role in the development of CRC.

Probiotics are live microorganisms that, when supplemented in equilibrate formulas, provide beneficial effects to the host. They are primarily lactic acid bacteria from genera such as *Lactobacillus*, *Streptococcus*, *Enterococcus*, *Lactococcus*, *Leuconostoc*, *Bifidobacterium*, and *Saccharomyces*. The therapeutic and preventive effects of probiotics are largely due to their ability to maintain a state of eubiosis, ensuring intestinal health. Probiotics help maintain gut health by protecting the intestinal mucosa, reducing the release of carcinogenic and oxidative molecules, and modulating immune responses, inflammation, autophagy, apoptosis, and cell differentiation. They also compete with harmful bacteria for nutrients and space, producing antimicrobial substances that limit the proliferation of pathogenic microbes, thereby preventing dysbiosis. The implementation of probiotics in CRC therapy is not only related to their ability to reduce the risk of CRC development and progression but also their potential to synergize with immunotherapy and chemotherapy while decreasing their unpleasant side effects [200]. In conclusion, the supplementation of probiotics pre- and/or post-surgery may improve prognosis and quality of life and reduce the recurrence of CRC [153,154,201]. In CRC patients, adjuvant therapy with probiotics decreases the occurrence of septicemia, the incidence of post-operative infections and diarrhea, and the rate of post-operative antibiotic use.

Yet, it is to be stressed that, despite the promising and encouraging results, not all probiotics showed positive health effects in CRC patients, emphasizing the need for further investigations. However, the in vitro and in vivo evidence paves the way for future translational research and development of probiotic- and postbiotic-based therapeutics for more personalized and targeted treatments of colorectal cancer.

## Figures and Tables

**Table 1:** List of preclinical research and clinical trials of the beneficial effects of probiotics administration in colorectal cancer management. The table reports an overview of the main probiotic strains tested in preclinical and clinical research, focusing on the molecular mechanisms of action, the dosage and the duration of the intervention, study design (experimental model for preclinical studies and inclusion criteria for clinical settings), and a summary of the advantages and current limitations (in term of study design, safety concerns, interaction with chemo/immunotherapy, lack of mechanistic insights). N/A, not applicable.

Probiotic Strain	Effects and Proposed Mechanism	Preclinical Evidence (Experimental Model) + [Ref]	Clinical Trial (Type, Sample Sizes) + [Ref]	Comments (Dosage, Treatment Durations, Patient Demographics)	Pros/Cons Limitations
*Lactobacillus rhamnosus* GG	Enhancement of gut barrier integrity	In vitro CRC model: high adhesion, high resistance against gastric acidity, and high antimicrobial activity against pathogens [175]	**NCT00197873** Prospective, multicenter, randomized, double-blind, placebo-controlled study N = 84	Dose: 20 × 10^9^CFU/daily Duration: 9 weeks/phase (lactobacilli/placebo), twice daily Subjects: advanced CRC patients undergoing chemotherapy (capecitabine, oxaliplatin, and bevacizumab)	Safety concerns related to immune suppression in cancer patients Potential interactions with chemotherapy
*Bifidobacterium longum* BB536 + *Lactobacillus acidophilus* LA1	Enhancement of mucosal immunity Reduction in pathogen adherence to the intestinal barrier	In vitro *CRC* model: LA1 boosts the immune system and balances intestinal microflora [176,177,178,179]	**NCT00936572** Prospective, randomized, double-blind, parallel-arm trial with three groups (high dose, low dose, placebo) N = 33 [180]	Dose: High = 10^9^ CFU, Low = 10^7^ CFU Duration: 3×/day from pre-operative day −5 to −1 and post-operative day 3 to 8 Subjects: CRC surgery patients (laparoscopy/laparotomy)	Small sample size Surrogate endpoints (colonization vs. clinical outcomes) and limited duration Unclear long-term safety Not powered to detect rare adverse events No stratification by microbiome baseline or immunotherapy
HEXBIO^®^ formulation *Lactobacillus acidophilus*, *L. lactis*, *L. casei*, *Bifidobacterium longum*, *B. bifidum*, *B. infantis*	Reduction in pro-inflammatory cytokines (IL-1β and IL-6) Upregulation of tumor suppressor miRNAs (miR-145 and miR-15a) Downregulation of oncomiRNAs (miR-21 and miR-155)	In vivo CRC mouse model: *B. longum* counteracts systemic inflammation, results in a drop in the aberrant crypt foci number in CRC mice and increased necrosis and fibrosis [47]	**NCT04021589** Randomized, double-blind, placebo-controlled study N = 60	Dose: 30 × 10^9^ CFU/day (twice daily) Duration: 6 months, initiated 4 weeks after surgery Subjects: CRC patients planned for colorectal resection	Robust trial design No major safety concerns reported Moderate sample size Delayed probiotic administration post-surgery Interaction with chemotherapy/antibiotics not directly analyzed
Colon Dophilus™ *Bifidobacterium breve* HA-129 (25%), *Bifidobacterium bifidum* HA-132 (20%), *Bifidobacterium longum* HA-135 (14.5%), *Lactobacillus acidophilus* HA-122 (8%), *Lactobacillus casei* HA-108 (8%), *Lactobacillus plantarum* HA-119 (8%), *Streptococcus thermophilus* HA-110 (6%), *Lactobacillus brevis* HA-112 (2%), *Bifidobacterium infantis* HA-116 (0.5%)	Decrease the activity of intestinal beta- D-glucuronidase	N/A	**NCT01410955** Randomized, quadruple-blind, placebo-controlled study, parallel assignment N = 46 [181]	Dose: 10 × 10^9^ CFU of bacteria (thrice per day) Duration: 12 weeks during irinotecan-based chemotherapy Subjects: patients with histologically proven CRC starting therapy with irinotecan	Varied irinotecan-based regimens Reduced sample size and statistical power Potential known and unknown confounders Lack of compliance assessment
Probio-Tec^®^ BG-VCap-6.5 *Bifidobacterium animalis* subsp. *lactis* BB-12^®^ + *Lactobacillus rhamnosus* GG (LGG^®^)	Competition for substrates with pathogenic bacteria, producing bacteriocins and enhancing transepithelial resistance Production of SCFAs Reduction in intestinal beta-D-glucuronidase activity	N/A	**NCT02819960** Phase 3 Multicenter, randomized, double-blind, placebo-controlled study N = 233 [182]	Dose: 2.7 × 10^9^ CFU of bacteria in 50%/50% ratio LGG^®^:BB-12^®^ (thrice per day) Duration: 6 weeks during chemotherapy Subjects: patients starting a new line of irinotecan-based therapy	Large sample size and well-powered randomized controlled trial Pooled analysis supports probiotic benefit in colostomy patients Limited by formula heterogeneity No compliance or biomarker data
Floratil *Saccharomyces boulardii*	Enhancement of mucosal immunity Maintenance of the intestinal epithelial barrier Inhibition of EGF-induced proliferation and increased apoptosis	In vivo Apc (Min) CRC mouse model: reduced tumor number and volume by 50% [183,184]	**NCT01609660** Phase 4, randomized, open-label, parallel-group trial N = 33	Dose: 100 mg/day orally Duration: 7 days pre-operatively Subjects: patients undergoing elective CRC resection	Targeted pre-operative use Small sample size Short administration period No follow-up beyond surgery
Synbiotic Forte™ *Pediococcus* *pentosaceus* 5-33:3, *Leuconostoc mesenteroides* 32-77:1, *Lactobacillus paracasei* ssp. *paracasei* 19, and *Lactobacillus plantarum* 2362	N/A	N/A	**NCT01479907** Double-blinded, prospective, randomized N = 100 [185]	Dose: 10^11^ of each of 4 LAB strains/dose (12 g in 250 cc of water) Duration: 15 days Subjects: patients who have undergone colectomy for cancer	Improved GI function and quality of life Short intervention duration Lack of microbiota or biomarker data
ProBion Clinica *Bifidobacterium lactis* Bl-04 (ATCC SD5219), *Lactobacillus acidophilus* NCFM (ATCC 700396)	Epigenetic changes	In vivo CRC rodent model: NCFM attenuates tumor growth by reducing levels of pro-carcinogenic metabolites in the gut [186]	**NCT03072641** Randomized, parallel assignment N = 20 [187]	Dose: tablets of 1.4 × 10^10^ CFU of Bl-04 + 7 × 10^9^ CFU of NCFM Duration: Avg. 31 ± 28 days pre-operatively (range: 8–78 days) Subjects: patients with at least one malignant tumor in the colon	Preliminary findings Lack of metabolomic insights Require validation in larger cohorts
HEAL 19 *Lactobacillus plantarum*	Decrease the expression of inflammatory markers Induction of cell cycle arrest and apoptosis Increased fecal IgA secretion	In vivo CRC model: nano-sized *L. plantarum* reduces colonic tumorigenesis, promotes lower colon weight/length ratios, and prevents animal weight loss [188]	**NCT03420443** Randomized, parallel assignment, triple-blind N = 30 [189]	Dose: control group: no treatment; oat bran group: 45 g of oat bran and freezing medium, synbiotic group: HEAL 19 (10^10^ CFU/g), freeze-dried blueberry husks (13 g), and oat bran (22 g) Duration: 2 weeks (1 pre-radiotherapy and 1 during radiotherapy) Subjects: patients with rectal adenocarcinoma	Feasibility and standardized baseline screening Small sample size Absence of dietary intake data Short intervention duration Lack of mechanistic insights
MIRACle study *Streptococcus thermophilus; Bifidobacterium breve; Bifidobacterium longum; Bifidobacterium infantis*; Lactobacillus acidophilus; Lactobacillus plantarum; Lactobacillus paracasei; Lactobacillus delbrueckii subsp. *Bulgaricus*	N/A	N/A	**NCT05164887** Case-control, prospective N = 131 [190]	Dose: Pre-, intra- (intraluminal anastomotic), and post-operative administration of probiotics (4.4 g equal to 450 × 10^9^ live bacterial cells/every 12 h) Duration: from day −5 until day +4 Subjects: CRC patients undergoing laparoscopic resections with ileo-colorectal anastomoses	Low complication rates despite worse baseline status support clinical consistency and feasibility of the approach Lack of randomization Absence of microbiota analysis limits generalizability and Lack of the mechanistic insights
* Lactobacillus acidophilus, Lactobacillus acidophilus * LA-EPS-20079, and *L. acidophilus* MTCC 5401	Decrease the expression of the inflammation-associated genes (TNF-α, IL-6, COX-2, iNOS) Increase the levels of SOD and catalase Promote apoptosis and inactivation of NF-kB pathway Increase in Th1 lymphocytes, decrease in Treg, and inhibition of M2 macrophages polarization	In vivo inflammation-driven CRC rodent model: reversion of the inflammation-induced colonic histological alterations and restoration of colonic permeability [191] In vivo mouse model: cytotoxic effects on cancer cells [192] In vivo mouse model: enhance the anti-tumor activity of CTLA-4 mAb therapy [193]	N/A	N/A	N/A
*Lactobacillus acidophilus* + *Bifidobacterium animalis* subsp. *lactis*	Decrease the activity of antioxidant enzymes (SOD) Increase the expression of apoptosis-related proteins (caspase-3 and Bax/Bcl-2 ratio)	In vivo CRC rodent model: inhibition of pre-neoplastic lesions [194]	N/A	N/A	N/A
*Lactobacillus acidophilus* CL1285 *+ Lactobacillus casei* LBC80R + *Lactobacillus rhamnosus* CLR2	Stimulation of quinone reductase activity	In vitro CRC model: antioxidant activity and inhibition of HT-29 cell proliferation [195]	N/A	N/A	N/A
* Lactobacillus casei * BL23	Downregulation of IL-22 Upregulation of caspase-7, caspase-9, and Bik	In vivo CRC mouse model: reduce histological scores and proliferative index values [196]	N/A	N/A	N/A
OMNi-BiOTiC^®^ 10AAD *Lactobacillus rhamnosus* MD14	Reduce fecal pro-carcinogenic enzymes Downregulation of oncogenic pathways (K-Ras, Wnt/β-catenin, NF-κB) Upregulation of p53	In vivo CRC rodent model: attenuated early colon carcinogenesis and aberrant crypt foci, restoring to almost normal colon histology [197]	**NCT03705442** Prospective, randomized, parallel assignment, placebo-controlled study, double-blind N = 76	Dose: two capsules (5 × 10^9^ CFU/each) per day, every 12 h Duration: 84 days (six chemotherapy cycles every 14 days) Subjects: histologically confirmed diagnosis of CRC with metastasis, patients starting first line of chemotherapy (FOLFIRI protocol)	Restoration of intestinal permeability Preliminary findings Requires validation in larger cohorts Absence of microbiota analysis limits generalizability and mechanistic insights
